# The Clinical Investigation Unit, Ham Green Hospital

**Published:** 1984-07

**Authors:** Angela M. Shepherd


### The Clinical Investigation Unit, Ham Green Hospital

**Angela M. Shepherd, M.D., M.R.C.O.G.**

In 1971 a small research unit was established in the corner of the respiratory function laboratory, attached to the Intensive Care Ward at Ham Green Hospital. The project was to study the problems of bladder control in both men and women by using objective measurements. Thus urodynamic techniques were introduced to Bristol by Roger Feneley, supported by the late Professor Atholl Riddell who seconded a research registrar for the first year.

The immediate task was to establish a method of investigating bladder disorders by measurement of urine flow rates and bladder and urethral pressures during filling and voiding. It is these tests which have become recognised as Urodynamic Studies and are accepted as an essential contribution to the investigation of the lower urinary tract. Reliable and interesting information was obtained within the course of the next few years and a number of research fellows published these and gave papers on their work undertaken in the Unit.

In 1977 the Medical Research Council gave us a substantial 5 year grant to study various aspects of lower urinary tract disorders. This included the continuation of urodynamic assessment, an evaluation of methods of clinical management and further work on aetiology and treatment of specific bladder conditions. We were asked to design a simple piece of equipment for use in urodynamic investigation and to join with the Medical Research Unit at Northwick Park Hospital in a study on the prevalence of incontinence in the community.

The Clinical Investigation Unit was thus established. More accommodation was urgently needed and we moved into the recently vacated Intensive Care Unit (I and J Wards). Our medical staff increased to include an Associate Specialist, a Clinical Assistant and two Research Fellows. Nurses, technicians, secretaries and physiotherapists completed the team and currently there are five full time and nine part time members contributing to the work of the Unit.

Following the completion of the studies supported by the M.R.C. we had established our reputation both nationally and internationally. Southmead Health Authority and the South Western Health Authority now provide financial support and we have undertaken a regional commitment. Our four main interests are those of clinical investigation and management of men and women with lower urinary tract symptoms, research into many aspects of lower urinary tract dysfunction and the dissemination of our acquired knowledge to nurses, doctors, paramedicals and to the public.

An ever increasing number of patients are referred to the Unit for urodynamic studies. We have seen nearly 9000 patients and now undertake studies on over 1000 new patients every year. Referrals are from all the specialties as well as directly from family doctors and from nurses. Patients travel from all three of the districts within Bristol as well as from Bath, Gloucester and Cheltenham and many centres in the South Western Region. Our tests include urinalysis, urine flow studies, urethral profilometry, urethral stress and sensitivity tests, filling cystometry, provocative tests for detrusor instability, voiding cystometry, measurement of pelvic floor function, anal sphincter and perineal electromyography, using both single fibre and concentric techniques and video-cysto-urethrography.

Two outpatient sessions are held every week rotating between B.R.I., Ham Green and Southmead Hospitals. Three nursing clinics and one catheter clinic are run every week by nurses with special expertise. Young and elderly patients with intractable urinary problems are admitted to the ward for a 5-day assessment and are managed by a team of ward nurses, physiotherapy and medical personnel. Surgery is undertaken when necessary. The conservative methods of management include drug therapy, pelvic floor re-education, bladder training, catheter management and advice on appropriate appliances, pads and pants. An interesting development has been that of the simple technique of intermittent self-catheterisation. This has been well documented in children with spina bifida but also has a useful application for men and women with underactive bladders and those with detrusor-sphincter dyssynergia occurring in neurological conditions such as multiple sclerosis. Management must be tailored to suit the individual and we have experts in all fields who are able to advise personally or by our telephone advisory service.

Although the regional clinical investigation and management commitment has increased greatly our research continues. Many aspects of urinary tract dysfunction have been studied in detail. Six postgraduate theses have been accepted and there are five more to be presented. Recently the role of endogenous opioids in the control of the lower urinary tract has been examined and the pathophysiology of the anal sphincter and pelvic floor in stress incontinence has been studied using the single fibre techniques in electromyography. Ongoing work includes studies on acute retention, prostatic carcinoma, incontinence in social service homes, the development of a microcomputer in urodynamic studies and the effect of traumatic vaginal deliveries on pelvic floor function.

A great variety of teaching has been undertaken by all the staff. During 1983 thirty two nurses' lectures were given and a two week residential course is held at 6 monthly intervals. Thirteen study days on urinary incontinence were held and members gave five guest lectures. Sixty-one doctors visited the Unit and thirty-two trainees from all over the world (doctors, nurses and technicians) stayed to learn our techniques.

Roger Feneley has directed the Unit throughout its development.

With the appointment of Paul Abrams as Consultant Urologist we have another dynamic director and plans for the future are tumbling over each other. It is hoped to upgrade the building to have facilities to computerise our own data, to improve our radio-diagnostic techniques and to enlarge our incontinence service to the community. We are in the 'plumbing business' to help all those who suffer the indignity of urinary incontinence, to cure those for whom it is possible, and to help to contain it for others so that they can remain socially acceptable and maintain their independence.

